# Impact of bovine respiratory disease on the pharmacokinetics of danofloxacin and tulathromycin in different ages of calves

**DOI:** 10.1371/journal.pone.0218864

**Published:** 2019-06-24

**Authors:** Danielle A. Mzyk, Claire M. Bublitz, Marilyn N. Martinez, Jennifer L. Davis, Ronald E. Baynes, Geof W. Smith

**Affiliations:** 1 Department of Population Health and Pathobiology, North Carolina State University College of Veterinary Medicine, Raleigh, North Carolina, United States of America; 2 Office of New Animal Drug Evaluation, Center for Veterinary Medicine, Rockville, Maryland, United States of America; 3 Department of Biomedical Sciences and Pathobiology, Virginia-Maryland College of Veterinary Medicine, Blacksburg, Virginia, United States of America; Plum Island Animal Disease Center, UNITED STATES

## Abstract

Pneumonia is one of the most economically important respiratory diseases of calves and knowledge of the impact of clinical disease on pharmacokinetics (PK) in young calves is limited. This study was undertaken to investigate the efficacy and PK of two antibiotics, tulathromycin and danofloxacin, in two age groups of calves experimentally infected with *Pasteurella multocida*. Both danofloxacin, a fluoroquinolone antibiotic, and tulathromycin, a macrolide antibiotic is approved for the treatment of bovine respiratory disease (BRD). To evaluate potential influences of age and disease on drug distribution and elimination in calves, plasma, interstitial fluid (ISF), and pulmonary epithelial lining fluid (PELF) were analyzed for drug concentrations. Concentrations for both drugs in the PELF were estimated by a urea dilution assay of the collected bronchoalveolar lavage fluids. Age was determined to be a significant covariate for calves administered danofloxacin and tulathromycin for plasma PK parameters. For calves administered danofloxacin, the area under the curve (AUC) in the plasma was lower in 6-month old calves (18.9 ± 12.6 hr* μg/mL) vs. 3-week old calves (32.0 ± 8.2 hr* μg/mL). Clearance (CL/F) of danofloxacin was higher in 6-month old calves. In contrast, tulathromycin plasma concentrations were higher in 6 month old calves and CL/F was higher in 3-week old calves. Age did not significantly influence the ISF concentrations of danofloxacin or tulathromycin in calves with respiratory disease, unlike previous studies which reported higher ISF concentrations of danofloxacin and tulathromycin in 6-month old calves when compared to younger calves. PELF concentrations were higher than plasma and ISF for both danofloxacin and tulathromycin, but did not differ between age groups. Potential reasons for age-related differences on plasma concentration–time profiles and the impact of disease on the partitioning of the drug from the blood to the lungs and ISF as a function of age are explored.

## Introduction

Pneumonia is one of the most economically important respiratory diseases of calves [1]. Its etiology is complex and can involve viruses, mycoplasmas and bacteria [2]. Bacteria, particularly *Pasteurella* spp., play an important role in many outbreaks of calf bronchopneumonia [3]. *Pasteurella multocida* (*P*. *multocida*), a Gram-negative bacteria, can increase the severity of the primary lung damage caused by viruses and exacerbate the clinical signs. Furthermore, studies indicate that *P*. *multocida* can act as a primary pathogen, producing severe acute pneumonia in calves [4, 5]. Due to the high prevalence of Gram-negative bacteria implicated as significant primary pathogens, the treatment of bovine respiratory disease (BRD) in cattle generally includes the use of Gram-negative spectrum antibiotics.

For this study, these drugs were selected for two reasons. First, they target different essential components of bacterial metabolism. Fluoroquinolones act by inhibiting DNA synthesis by targeting the activity of both DNA gyrase and the topoisomerase IV enzymes. Macrolides inhibit bacterial protein synthesis by targeting various ribosomes and damaging critical bacteria proteins. Second, both drug classes represent different mechanisms of eradication of bacterial infections. Macrolides are considered to be one of the classic bacteriostatic drugs, which prevent bacterial growth, but not killing bacteria. The efficacy of macrolides have been evaluated by the the time above the minimum inhibitory concentration (MIC). Quinolones are generally “bactericidal” meaning it kills bacteria. For fluoroquinolones, two pharmacokinetic/pharmacodynamic indices are correlated with the effectiveness of an antimicrobial treatment: area under the curve//MIC and maximum plasma concentration/MIC. In practice, efficacy based solely on these classifications can lead to false assumptions of clinical efficacy, especially if other major PK/pharmacodynamic parameters are ignored. Although the main goal of treatment with either type of antibiotic is to eradicate infection, several host related factors can influence the efficacy of these treatments in calves. Age-related changes in cattle, including body composition, metabolism and clearance mechanisms as well as the competency of the immune response can affect the efficacy of these drugs.

Fluoroquinolones, such as danofloxacin, have various pharmacologic properties of that contribute to the clinical success of treating BRD. These properties include quick times to maximum concentrations (T_max_), high maximum plasma concentrations (C_max_), large volumes of distribution (Vd, Vd/F) and excellent penetration in lung fluid with rapid bactericidal activity against commonly isolated Gram-negative bacterial respiratory pathogens [6–8]. Since a majority of bacterial infections take place in the extracellular fluid of tissues, the drug concentration in this compartment determines the efficacy of an antibiotic.

Macrolide antibiotics, including tulathromycin, accumulate in leukocytes and bronchial secretions, and are present at concentrations in lung tissues that markedly exceed concentrations in the plasma [9]. Clinical disease and inflammation has been shown to influence the PK of tulathromycin. In pigs with induced respiratory infections, tulathromycin concentrations in tissues and the elimination half-life increased as compared to that observed in healthy controls [10]. Tilmicosin, another macrolide used in veterinary medicine, has been shown to accumulate in significantly higher concentrations in the lungs of rats with respiratory disease than healthy rats, although serum concentrations were not different between the two groups [11]. However, the increase in pulmonary concentrations of macrolides in the presence of disease is not a universal phenomenon as other macrolides, such as erythromycin, have shown a decrease in penetration into the lung tissue of calves with clinical respiratory disease [12].

Clinical disease has also been shown to alter the PK of other pharmacological compounds in cattle. In some situations, these PK changes may be linked to alteration in Phase I metabolizing enzymes and subsequent disease-associated changes in drug clearance [13]. Disease-associated changes in drug metabolism may or may not be confounded with age-associated differences in the enzyme maturation process, indicating a need to evaluate the distribution and PK of danofloxacin and tulathromycin in disease states in calves if we are to consider use of these compounds in pre-ruminating calves [14, 15]. Since these drugs are intended for use in calves over a large age range, it is imperative to understand the impact of infection and inflammation on the distribution of tulathromycin and danofloxacin in different age calves.

In the United States, danofloxacin is approved for treatment of BRD associated with *Mannhemia haemolytica* (*M*. *haemolytica*) and *P*. *multocida* when administered as a single dose of 8 mg/kg, SC. Tulathromycin is approved for treatment of BRD associated with *M*. *haemolytica*, *P*. *multocida*, and *Histophilus somnus* (single dose of 2.5 mg of tulathromycin/kg, SC). The purpose of the study reported here was to compare the concentrations of danofloxacin and tulathromycin in plasma, interstitial fluid (ISF) and pulmonary epithelial lining fluid (PELF) of calves treated with these antimicrobial drugs after challenge with *P*. *multocida*. By evaluating drug kinetics in affected calves of two different ages (3 weeks and 6 months), the concomitant influence of disease and age on the distribution and PK could be examined. A comparison of these results to outcomes previously reported in normal healthy 3 week and 6-month-old calves provides insights into whether disease influences the age-associated differences in the PK of these two compounds.

## Materials and methods

### Animals

This study was approved by the North Carolina State University Institutional Animal Care and Use Committee. Weaned and milk-fed male Holstein calves were bought from a local dairy herd. Eighteen milk-fed Holstein calves, 2–3 weeks of age, weighing between 45.5–70.5 kg were purchased and trailered to the treatment facility. Calves were housed in groups of two, fed commercial milk replacer twice a day, and allowed access to water and calf starter (Milk Specialties, Inc., Eden, MN) throughout the study. Eighteen weaned calves, 6 months of age and weighing between 188–281 kg at time of study, were purchased from a local dairy herd. The calves were group housed indoors on a concrete floor bedded with wood shavings, fed grass hay and allowed free access to water throughout the study. None of the calves had any previous history of disease or antibiotic administration and had normal physical examinations prior to start of the study. All calves were euthanized at the end of the study.

### Experimental infection

All calves were subjected to the physical stress of a two-hour trailer ride prior to being inoculated with the pathogen. On day 0, all calves were inoculated with field isolates of *P*. *multocida* from confirmed clinical cases via Collison nebulizer (Jorgenson Labs Inc. Loveland, CO). This device produces single-cell droplets, < 3 μm in diameter, delivered using a head mask fitted with an inhalation and exhalation port placed over the nostrils and mouth. The nebulizer delivered 10 mL of bacterial suspension containing approximately 1 x 10^9^ CFUs and the time of exposure lasted 20–25 min. Calves were monitored throughout nebulization with vitals taken every hour post nebulization to monitor clinical progress. The bacteria used in this study were shown to be susceptible to danofloxacin and tulathromycin. All susceptibility testing in our laboratory was conducted according to CLSI standards. Three isolates from clinical field strains of *P*. *multocida* were re-steaked from glycerol stocks onto trypticase soy agar supplemented with 5% sheep blood and incubated at 37C overnight. The next day, a single colony was selected and inoculated into 100 mL of tryptic soy broth. The broth was enriched for 18–20 hours at 37° C. After enrichment, a 1 mL aliquot was removed and serially diluted ten-fold, spot plated onto blood agar plates, and incubated to determine the starting concentration. The remaining enrichment was used to challenge calves.

#### Clinical score assessment

Each calf was assessed using a clinical score sheet from the University of Wisconsin Calf Respiratory Scoring System [16]. Briefly, this 12-point system assigns a score based on rectal temperature, nasal discharge, and eye and ear scores. Each criterion is graded on a 0–3 scale, with 0 being normal and 3 being the highest severity. Calves were enrolled into the trial that scored ≥ 5 after nebulization. The score of each calf was noted prior to induction and every 24 hours after drug administration.

#### Thoracic ultrasound

Ultrasonography was performed using an 8.5 MHz linear probe that was directly applied on the thorax after 70% isopropyl alcohol had been sprayed. All calves were scanned on each side of the thorax for the presence of abnormal ultrasonographic findings and scored based on previously validated scoring system [17]. Lungs were scanned prior to nebulization and every 24 hours throughout the study.

### Drug administration and blood collection

All calves were weighed on a digital scale on the morning of the study commencement for determination of the administered dose. Upon arrival, calves were restrained for intravenous catheter placement. The area where the catheter was to be placed was clipped and aseptically prepared. Using sterile technique, a 14 G x 3.25 inch catheter (Angiocath, BD, Franklin Lakes, NJ, USA) was inserted into the right jugular vein, an extension set was then attached and both were sutured in place using a 2–0 monofilament suture. Catheters were flushed three times a day using 6 mL of heparinized saline (10 units/ml). Depending on group assignment, a single SC injection of danofloxacin (8 mg/kg) (Advocin; Zoetis, Florham Park, NJ, USA) or tulathromycin (2.5 mg/kg) (Draxxin, Zoetis, Florham Park, NJ, USA) was administered to each calf in the neck per label instructions.

For calves administered danofloxacin, blood samples were collected from the jugular catheter and transferred to lithium heparinized tubes at time 0 (pretreatment), 0.25, 0.5, 1, 2, 4, 6, 8, 10, 12, 24, 36, 48, 60, 72, 84, 96, 108, 120, 132, and 144 hours post administration of the drug. For calves administered tulathromycin, blood samples were taken at 0, 0.25, 0.5, 1, 3, 4, 6, 8, 12, 24, 36, 48, 60, 72, 84, 96, 108, 120, 132, 144, 156, 168, 180, 192, 204, 216, 228, 240, 252, 264, 276, 288, and 300 hours post administration. These samples were stored on ice until centrifugation at approximately 3500 x *g* for 20 min to separate plasma. The plasma samples were stored at -80°C until analysis.

### Interstitial fluid collection

All calves were implanted with SC ultrafiltration interstitial fluid (ISF) probes on the side of the neck opposite the site of the SC drug injection (BASI Inc, West Lafayette, IN). Each probe contained three semi-permeable loops connected to a non-permeable tube that extended outside the animal and attached to a 3 ml no additive plastic vacutainer tube. This tube provided negative pressure for fluid collection through small pores in the probe membranes. These pores allowed for the movement of water, electrolytes and low molecular weight molecules (<30,000 Da) to pass into collection tube. This pore size excludes large molecules such as proteins, protein bound drugs, and cells. Probes were placed with calves under sedation with xylazine [Rompun Injectable (20mg/ml); Bayer Animal Health Division] at a dose of 0.05–0.1 mg/kg in the cervical neck muscles. Probes were placed twenty-four hours prior to the start of the trial to allow adequate time for equilibration with body fluids. One probe was inserted into each calf subcutaneously in the area cranial to the scapula. All probes were placed through a small stab incision using an introducer needle provided by the company. The ISF was collected at time 0 (pretreatment), 2, 4, 6, 8, 10, 12, 24, 36, 48, 60, 72, 84, 96, 108, 120, 132, and 144 h after SC administration of danofloxacin. For tulathromycin, ISF was collected at time 0 (pretreatment), 3, 4, 6, 8, 12, 24, 36, 48, 60, 72, 84, 96, 108, 120, 132, 144, 156, 168, 180, 192, 204, 216, 228, 240, 252, 264, 276, 288, and 300 hours. Since each ISF sample represents fluid collection over a certain amount of time (i.e. not instantaneous sampling), a lag time was calculated based on the length of the tube and fluid collected over time for each sample. The fluid collected was frozen at -80°C until analysis.

### Lung fluid collection

To determine drug concentrations in the PELF, a BAL was performed using a method described previously [18]. Briefly, BALs were performed in all 3-week old calves using a sterilized, flexible 10 French X 36 inch catheter with a 3-cc balloon cuff and in ruminating calves a 24 French X 59 inch catheter was used (Mila International, Inc. Medical Instrumentation for Animals, Florence KY). At each time point, the calf was restrained and the head and neck of the calf were extended to facilitate passage of the sterile BAL catheter. The BAL catheter was introduced into the ventral meatus of the nose through which it was advanced down the trachea until it was wedged in a terminal bronchus. Repeated coughing was used as an indicator of appropriate placement. In the wedged position, the balloon cuff was inflated to create a seal and the catheter was held firmly in place while the guide-wire was removed. At each time point, 100 ml of sterile saline were infused into the lungs. Immediately after the infusion, negative pressure was applied to aspirate fluid. The volume of fluid that was retrieved ranged from 4 to 75 ml of clear to mildly turbid foamy fluid. The fluid sample was placed into a sterile collection tube, the total amount recorded and placed on ice until centrifugation. The BAL samples were centrifuged at 300 x *g* for 10 min and supernatant fluid was separated from cell pellet and frozen at -80°C until analysis.

### Drug analysis

Total (protein bound and free) plasma and BAL fluids concentrations were determined as total tulathromycin and danofloxacin. ISF concentrations were solely the unbound concentrations of the two compounds. Plasma and BAL fluids were analyzed by either high-performance liquid chromatography (HPLC) with fluorescence detection (danofloxacin) or by ultra-performance liquid chromatography (UPLC) tandem mass spectrometry (MS/MS) (tulathromycin) following solid phase extraction. The ISF was injected directly onto the HPLC/UPLC. Danofloxacin was extracted from plasma and BAL supernatant using a modified enrofloxacin extraction method described previously [19]. For tulathromycin, plasma samples for UPLC-MS/MS analysis were pretreated by mixing 500-μL of plasma with 500-μL of 4% phosphoric acid, and vortexing for 10 seconds. The 1 mL pretreated sample was then loaded onto Oasis 3 cc (60 mg) PRiME HLB cartridges (Waters Corporation) and pulled through with a vacuum at ~3 psi. Each sample was then washed with 1 mL of 5:95 (v:v) methanol:water. Samples were then eluted from the cartridge using 400-μL of 60:40 (v:v) acetonitrile:water with 0.1% formic acid. The collected liquid was then transferred to a vial and 5-μL aliquot was analyzed by the UPLC-MS/MS.

Validation standards were prepared over a linear range for each matrix (plasma, ISF, sodium chloride 0.9% (as a substitute for PELF), mobile phase) and were used to construct calibration curves. These standards were validated over the range 0.001–5.0 μg/mL in fortified (spiked) blank plasma, BAL and ISF with danofloxacin or tulathromycin (reference drug standards for both danofloxacin and tulathromycin were provided by Zoetis) to validate the HPLC analysis.

The percent coefficient of variation (%CV) for inter and intra-day danofloxacin recovery averaged 10.7% (with an average recovery of 95.9%) over the validated range. The limit of quantification (LOQ) was 5 and 10 ng/ml and the limit of detection (LOD) was 1 and 5 ng/mL for plasma and BAL, and ISF respectively.

For tulathromycin, for each assay matrix, calibration curves were constructed spanning over the range of 5–1000 ng/mL. The R^2^ values for the calibration curves were 0.99. Intra and inter-day %CV were less than 20%, and the accuracy ranged from 102 to 106%. The LOQ was 5 ng/mL, with a precision of 8% and an accuracy of 105%.

#### Concentrations in PELF

Estimation of the amount of PELF sampled by BAL fluid was performed using the urea dilution method as described previously in cattle [20]. Urea nitrogen concentrations in serum and BAL fluid was determined by use of a urea test kit (Urea test kit; Sigma Chemical, St Louis, MO, USA) and the absorbance values measured by use of a spectrophotometer.

### Pharmacokinetic analysis

A compartmental PK analysis was generated using Phoenix 64, Certara, Princeton, NJ, USA. The influence of age and calf respiratory score on primary and secondary PK parameters was determined using a nonlinear mixed-effect model (Phoenix WinNonlin/NLME, Version 1.3 Certara). For both danofloxacin and tulathromycin, the population base model was fitted as a multiplicative two-compartment model parameterized by clearance. Model selection was based upon the precision of parameter estimates, goodness-of-fit plots (e.g., residual plots) and statistical significance between models using lowest log-likelihood ratio (-2LL) values obtained in the software. A preliminary compartmental analysis was conducted with Phoenix WinNonlin to obtain the initial estimates for the parameters of the basic model (i.e. no covariates).

For both danofloxacin and tulathromycin, a determination assessment of the contribution of age and respiratory score to calf pharmacokinetics was assessed by improvement in the objective function following the addition of each term to the base model. A box plot of effect of the covariate (age and respiratory score) on each parameter showed that clearance (CL) expressed as a function of bioavailability (CL/F) was the parameter most likely affected by age. Age was added to the base model as a categorical covariate (where 3-week old calves = 0 and 6 month old calves = 1), and its effect on the PK parameters, the apparent volume of distribution (Vd) expressed as a function of bioavailability (Vd/F) and Cl/F was evaluated using a likelihood ratio test. A *P*-value ≤ 0.05 was considered to be significant. For both tulathromycin and danofloxacin, age was found to significantly improve model predictions for CL/F (P < 0.001), but not Vd/F. After age was determined to improve the model, this covariate remained in the final model. Total respiratory score was determined to not be a significant covariate irrespective of calf age. All PK parameters, except for elimination half-life (T½), were reported as a geometric mean. The individual animal PK values were calculated, and the descriptive statistics (geometric mean ± SD) were reported. Goodness-of-fit plots for the final models are available in the supporting information (danofloxacin: S1a and S1b Figures; tulathromycin: S2a and S2b Figures).

Statistical comparisons of PK parameters from the compartmental analysis and measured clinical outcomes of each age group were performed with the Kruskal Wallis test for nonparametric data using SigmaPlot (Systat Software, Inc, San Jose, CA, USA); P-values of ≤0.05 using a two tailed test were considered statistically significant. The normality assumption was tested for each variable set with the Shapiro–Wilk W-test, which is the preferred method for testing the normality of data when the sample size is small [21]. PELF concentrations for all groups were averaged at all-time points.

## Results

Jugular catheters and ISF probes were well tolerated. During some sampling time intervals, fluid was not collected into the ISF probe, so ISF data is missing for these time points. One 3-week old calf (enrolled in the group administered tulathromycin) died 8 hours post nebulization. Necropsy results attributed the cause of death to significant pulmonary edema due to high endotoxin levels and *P*. *multocida* was cultured from lungs and joints. During BAL collection, harvested fluid volumes varied considerably.

### Clinical health scores and thoracic ultrasound scores

The mean clinical health and ultrasound scores, as well as rectal temperatures throughout the study are presented in S1 and S2 Tables. Prior to drug administration, the clinical health score and thoracic ultrasonographic score did not differ significantly between the two age groups. All calves prior to administration of either study drug attained a respiratory disease score ≥ 5. Scores were monitored throughout the study period. Total respiratory scores decreased throughout the study in both groups.

### Danofloxacin

#### Plasma

**Fig 1:**
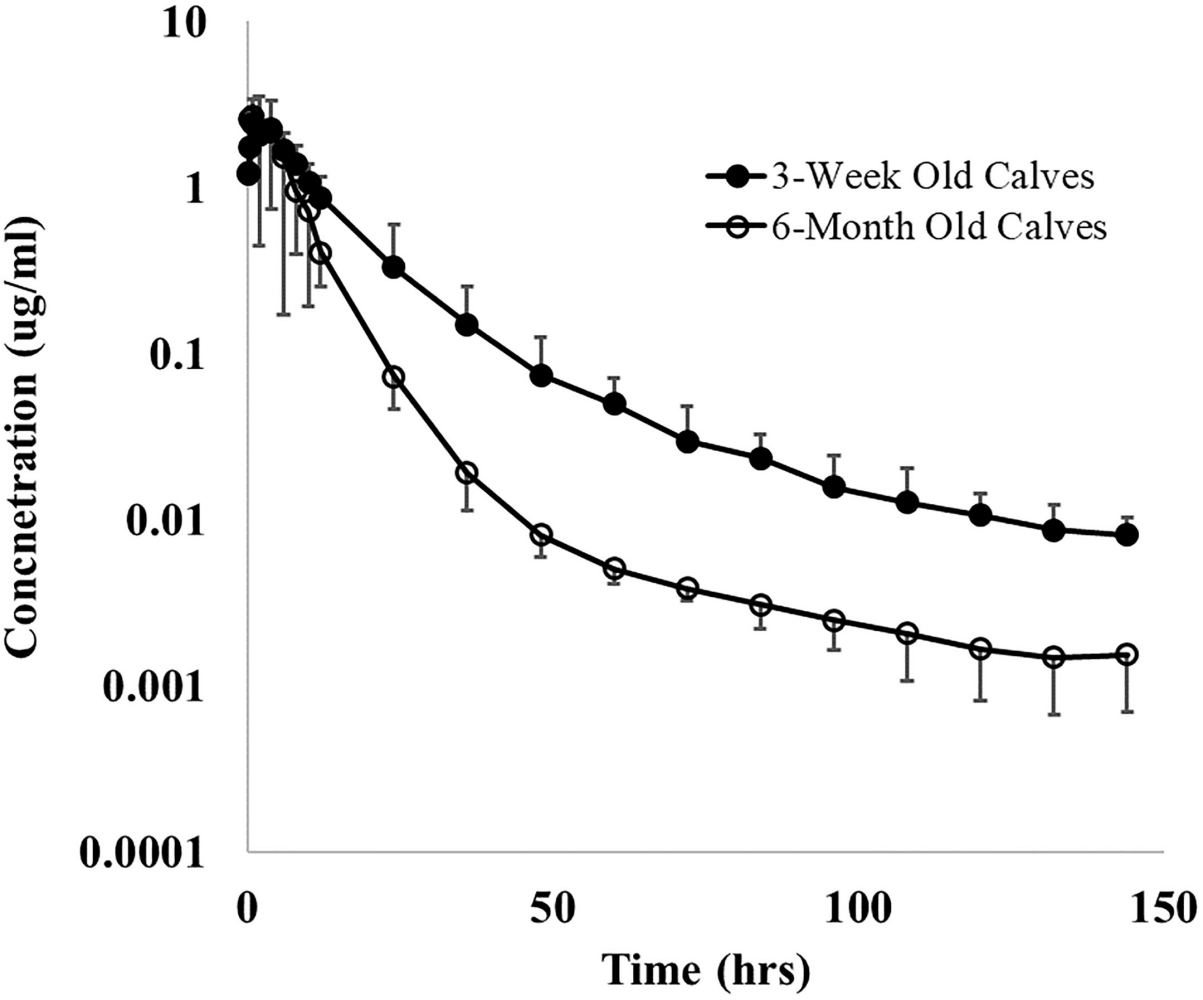
Mean (±SD) plasma concentrations of danofloxacin. Danofloxacin was administered by subcutaneous route at doses of 8 mg/kg in 3-week and 6-month old calves with induced respiratory disease from *P*. *Multocida*.

**Fig 2:**
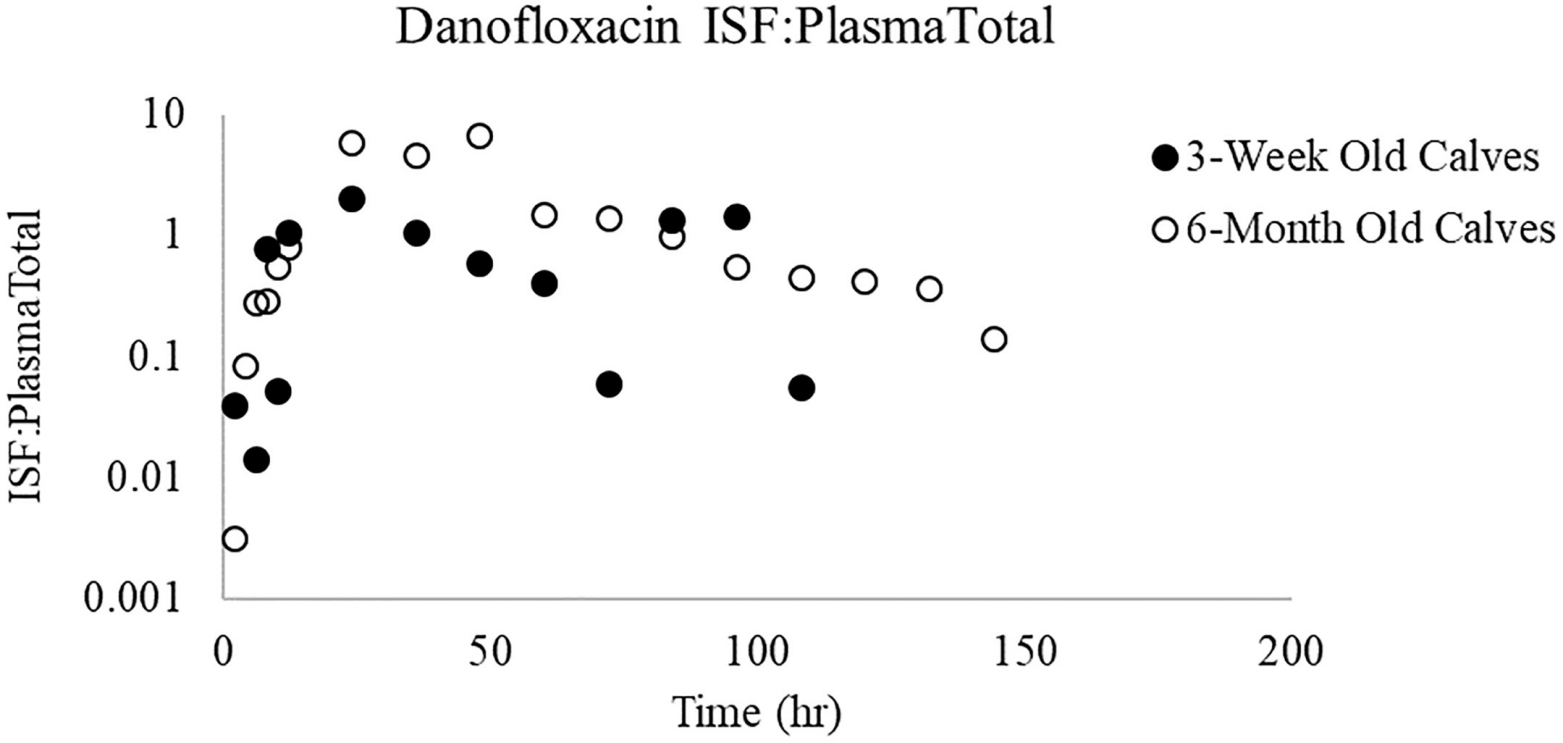
ISF:Plasma_Total_ ratios for danofloxacin. Ratios comparing unbound drug in ISF to total drug (bound and unbound) in plasma.

The fitted PK parameters of danofloxacin following a single 8 mg/kg SC danofloxacin in 3-week and 6-month old calves with respiratory disease are shown in Tables 1 and 2 and Fig 1. Danofloxacin demonstrated a Vd/F of 3.1 L/kg and 3.5 L/kg in 3-week old calves and 6-month old calves respectively. 3-week old calves had a significantly lower mean CL/F (262.9 ml/hr/kg) as compared to 6-month old calves (516.3 ml/hr/kg). However, these age-related differences in clearance were not reflected in plasma terminal elimination half-life (T½) values. The 3-week old calves presented with T½ similar harmonic mean (±harmonic SD) values of 34.45 ± 15.5 hours while that of the 6-month-old calves were 34.11 ± 20.1 hours. The area under the curve (AUC) was significantly greater in the 3-week old calves as compared to older calves. (Table 1). After SC administration, the maximum danofloxacin concentration in plasma (C_max_) reached a value of 2.4 μg/mL for 3-week old and 3.6 μg/mL in 6-month old calves. The mean time to maximum concentration (T_max_) was 2.4 and 2.0 h, respectively. Although ratios were variable, there was a trend for lower ISF:Plasma_Total_ ratios in 3-week old calves as compared to 6-month old calves (Fig 2).

**Table 1:** Pharmacokinetic parameters for danofloxacin in 3-week old vs. 6-month old calves with respiratory disease.

		3-Week Old Calves		6-Month Old Calves	
Parameter		Median	Range	Median	Range
AUC_inf_	hr*μg/ml	29.8*	23.3–49.6	14.2*	10.9–51.4
CL/F	ml/hr/kg	268.6*	161.3–342.2	561.6*	155.6–731.6
T_1/2_	hr	35.8	24.8–59.4	32.2	18.3–134.9
C_max_	μg/mL	2.2	1.7–4.2	1.5	1.1–10.4
T_max_	hr	2.0	0.8–3.1	1.4	0.4–3.7
V_d_/F	L/kg	2.9	1.8–4.0	3.4	0.7–5.9

*Indicates significantly different by *t*-test (*P* < 0.05).

**Table 2:** Population pharmacokinetic parameters for danofloxacin in 3-week old vs. 6-month old calves with respiratory disease.

Population Values—Final NLME Model				
Parameter	Units	Estimate	Stderr	CV%
*T*_MAX_	h	2.2	0.9	60.8
*C*_MAX_	μg/mL	3.0	1.4	79.4
θ*V*	L/kg	2.9	0.26	9.1
θ*V2*	L/kg	1.22	0.24	19.7
Cl	L/kg/h	0.26	0.017	6.9
Cl2	L/kg/h	0.03	0.0045	13.5
*T*_½_*	h	29.5	2.2	40.7

Population values from NLME model and median and range of PK parameters from compartmental analysis after single S.C. injection of 8 mg/kg danofloxacin in 3-week-old vs. 6-month-old calves with induced respiratory disease with *P*. *multocida*.

*Indicates significantly different by *t*-test (*P* < 0.01).

### Interstitial fluid

Using an *in vivo* ultrafiltration technique to collect ISF in repeated samples allowed for the monitoring of unbound drug disposition over time and was less invasive than tissue biopsies. Although some samples were missed due to occlusion of the probe, these devices were well tolerated and collected between 0.05 μL and >2 mL of fluid per sampling time for drug analysis.

**Fig 3:**
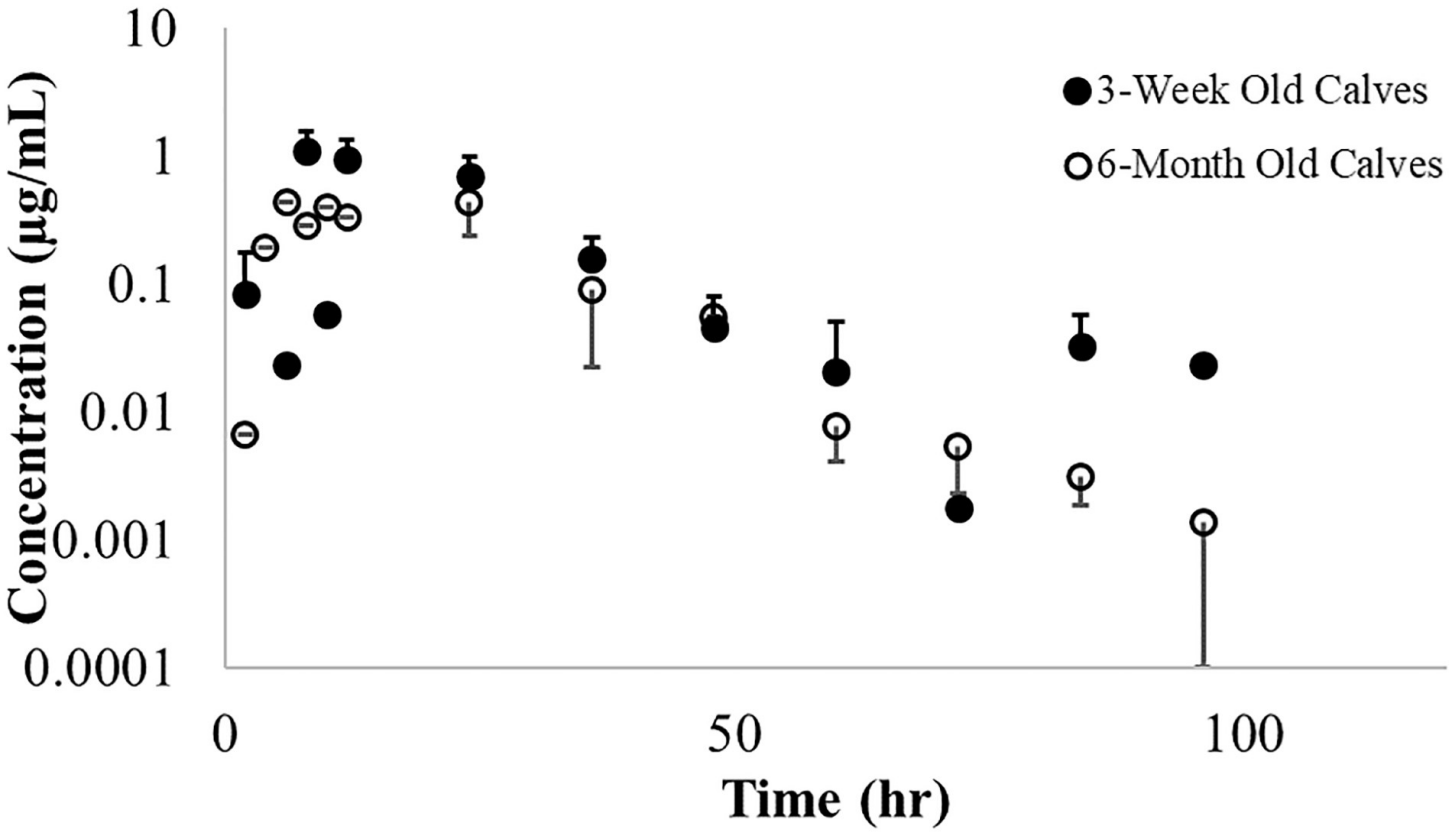
Mean interstitial fluid ± SD concentrations of danofloxacin. Danofloxacin was administered via single S.C. injection of 8 mg/kg danofloxacin in 3-week old (solid circles) and 6-month old calves (open circles).

The T_max_ for ISF fluid occurred later than in plasma irrespective of calf age. There was no difference in mean (±SD) concentration of danofloxacin in ISF samples from both groups of calves (Fig 3).

#### Pulmonary epithelial lining fluid

**Fig 4:**
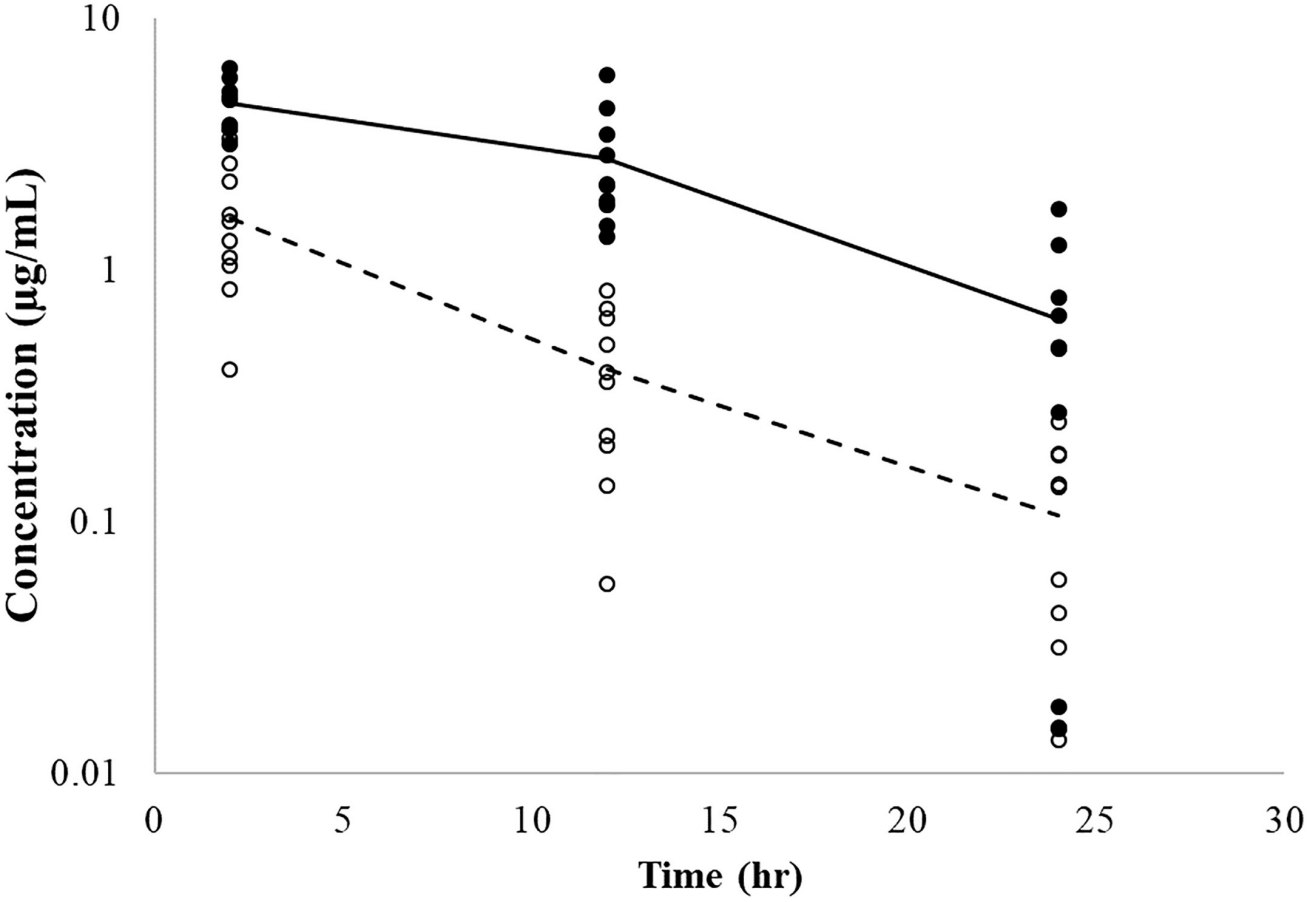
Individual PELF concentrations of danofloxacin. Lines represent average concentration over time for 3-week old calves (solid line) and 6-month old calves (dashed line).

The maximum concentration in PELF was noted to occur at the first sampling time (2 h post administration) in both groups, with estimated PELF concentrations far exceeding the concentrations seen in plasma and ISF (Fig 4). High variability in drug concentrations in PELF was seen in both groups of calves. Three week old calves showed trends for higher concentrations of danofloxacin in PELF as compared to 6-month old calves.

### Tulathromycin

#### Plasma

**Fig 5:**
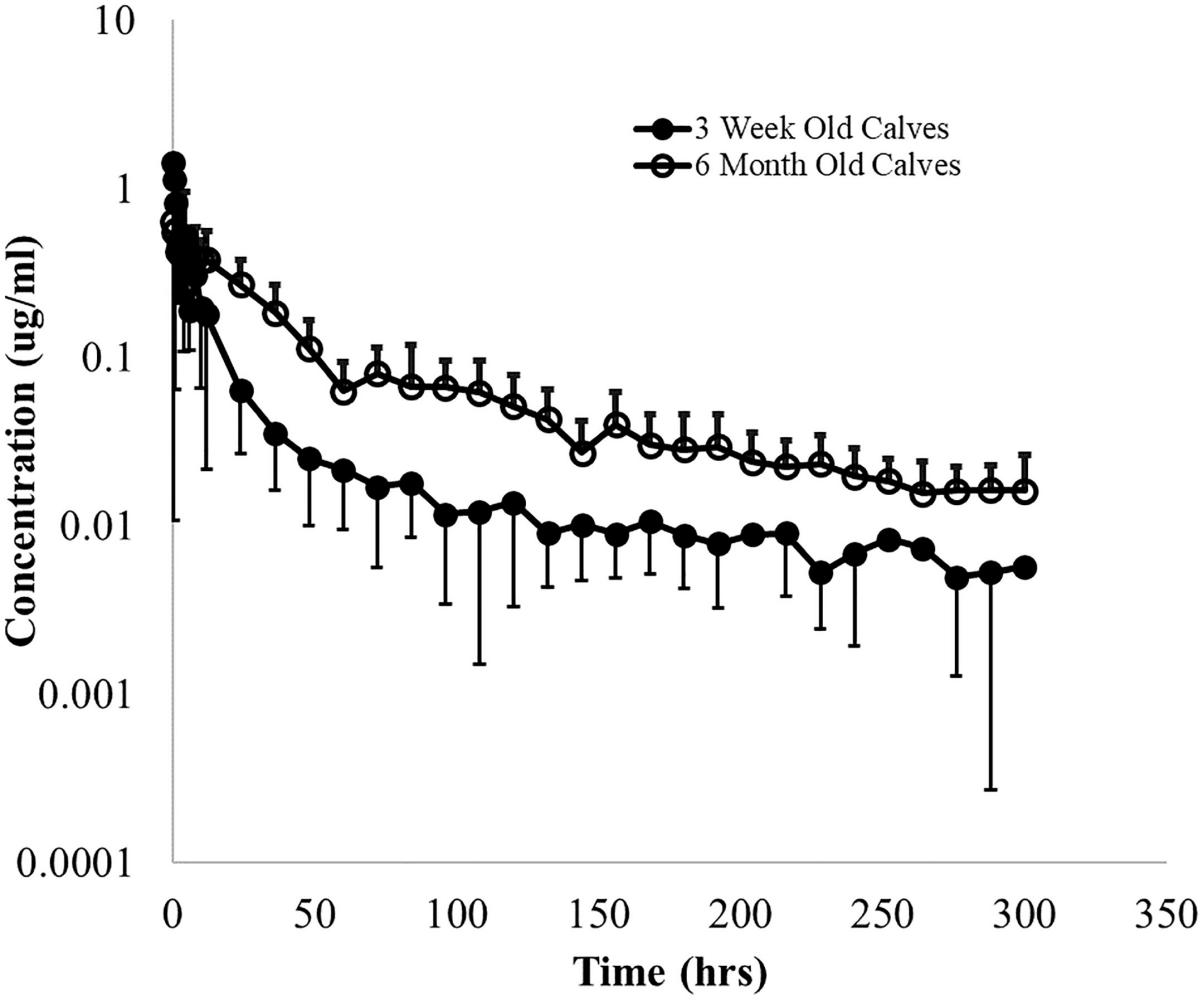
Mean (±SD) plasma concentrations of tulathromycin. Tulathromycin was administered by subcutaneous route at a dose of 2.5 mg/kg in 3-week and 6-month old calves with induced respiratory disease from *P*. *Multocida*.

**Fig 6:**
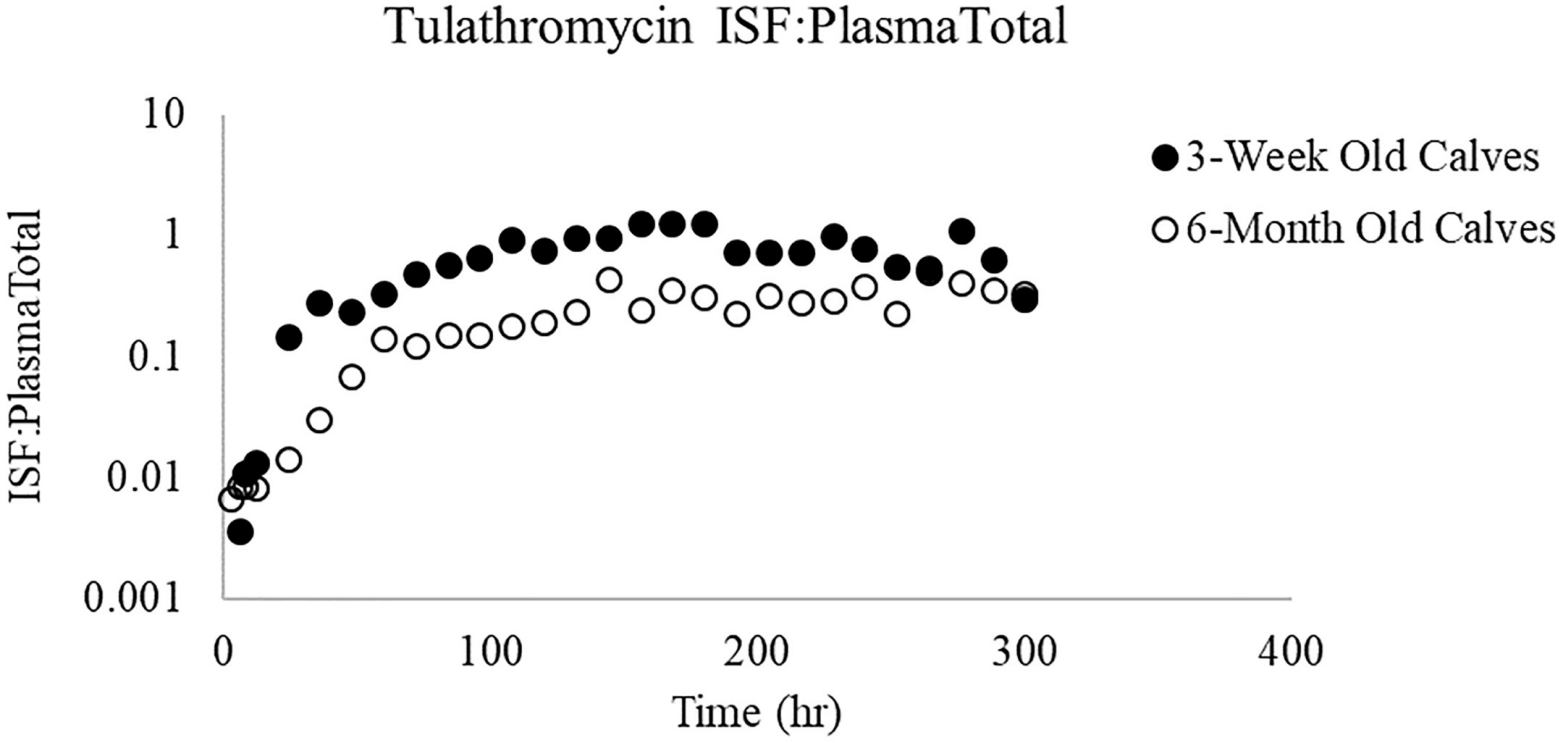
ISF:Plasma_Total_ ratios for tulathromycin. Ratios comparing unbound drug in ISF to total drug (bound and unbound) in plasma.

The PK parameters and plasma concentrations of tulathromycin in two different ages of calves affected by respiratory disease following SC administration of 2.5 mg/kg once are shown in Tables 3 and 4 and Fig 5 respectively. Following SC administration, tulathromycin demonstrated a Vd/F of 27.9 L/kg and 14.7 L/kg in 3-week old calves and 6-month old calves respectively. The CL/F values estimated for the 3-week old calves (343.3 ml/hr/kg) was significantly greater than that estimated in the 6-month old calves (126.5 ml/hr/kg). The harmonic mean plasma T½ ± harmonic SD was longer in 6-month old calves (114.9 ± 74.2 hours) as compared to that observed in the 3-week old calves (98.7 ± 52.6 hours). The AUC was significantly lower in the 3-week old calves as compared to that estimated in the older calves (Table 2). After SC administration, the maximum tulathromycin concentration in plasma (C_max_), reached a value of 0.49 ± 0.26 μg/mL for 3-week old and 0.54 ± 0.53 μg/mL for the 6-month old calves. The corresponding mean T_max_ values were significantly later in 6-month old calves (3.4 h) than in the 3-week old calves (0.6 h). Although ratios were variable, there was a trend for higher ISF:Plasma_Total_ ratios in 3-week old calves as compared to 6-month old calves (Fig 6).

**Table 3:** Pharmacokinetic parameters for tulathromycin in 3-week old vs. 6-month old calves with respiratory disease.

		3-Week Old Calves		6-Month Old Calves	
Parameter		Median	Range	Median	Range
AUC_inf_	hr*μg/ml	6.7*	4.7–27.1	20.6*	11.9–30.2
CL/F	ml/hr/kg	373.3*	92.3–534.8	121.3*	82.8–210.6
T_1/2_	hr	116.5	50.8–729.1	91.6	51.6–375.7
C_max_	μg/mL	0.4	0.3–1.1	0.3	0.3–1.8
T_max_	hr	0.5*	0.2–1.3	2.1*	0.3–7.3
V_d_/F	L/kg	25.9	11.3–63.7	9.9	2.5–16.7

*Indicates significantly different by *t*-test (*P* < 0.05).

**Table 4:** Population pharmacokinetic parameters for tulathromycin in 3-week old vs. 6-month old calves with respiratory disease.

Population Values—Final NLME Model				
Parameter	Units	Estimate	Stderr	CV%
*T*_MAX_	h	2.0	1.6	109.7
*C*_MAX_	μg/mL	1.3	0.9	81.5
θ*V*	L/kg	3.7	1.06	55.0
θ*V2*	L/kg	19.6	2.9	71.5
θ*V3*	L/kg	1.3	0.56	49.9
Cl	L/kg/h	0.20	0.30	67.8
Cl2	L/kg/h	0.24	0.38	78.2
CL3	L/kg/h	1.03	0.12	11.5
*T*_½_*	h	100.3	7.0	69.8

Population values from NLME model and median and range of PK parameters from compartmental analysis after single S.C. injection of 2.5 mg/kg tulathromycin in 3-week-old vs. 6-month-old calves with induced respiratory disease with *P*. *multocida*.

* Indicates significantly different by *t*-test (*P* < 0.05).

#### Interstitial fluid

**Fig 7:**
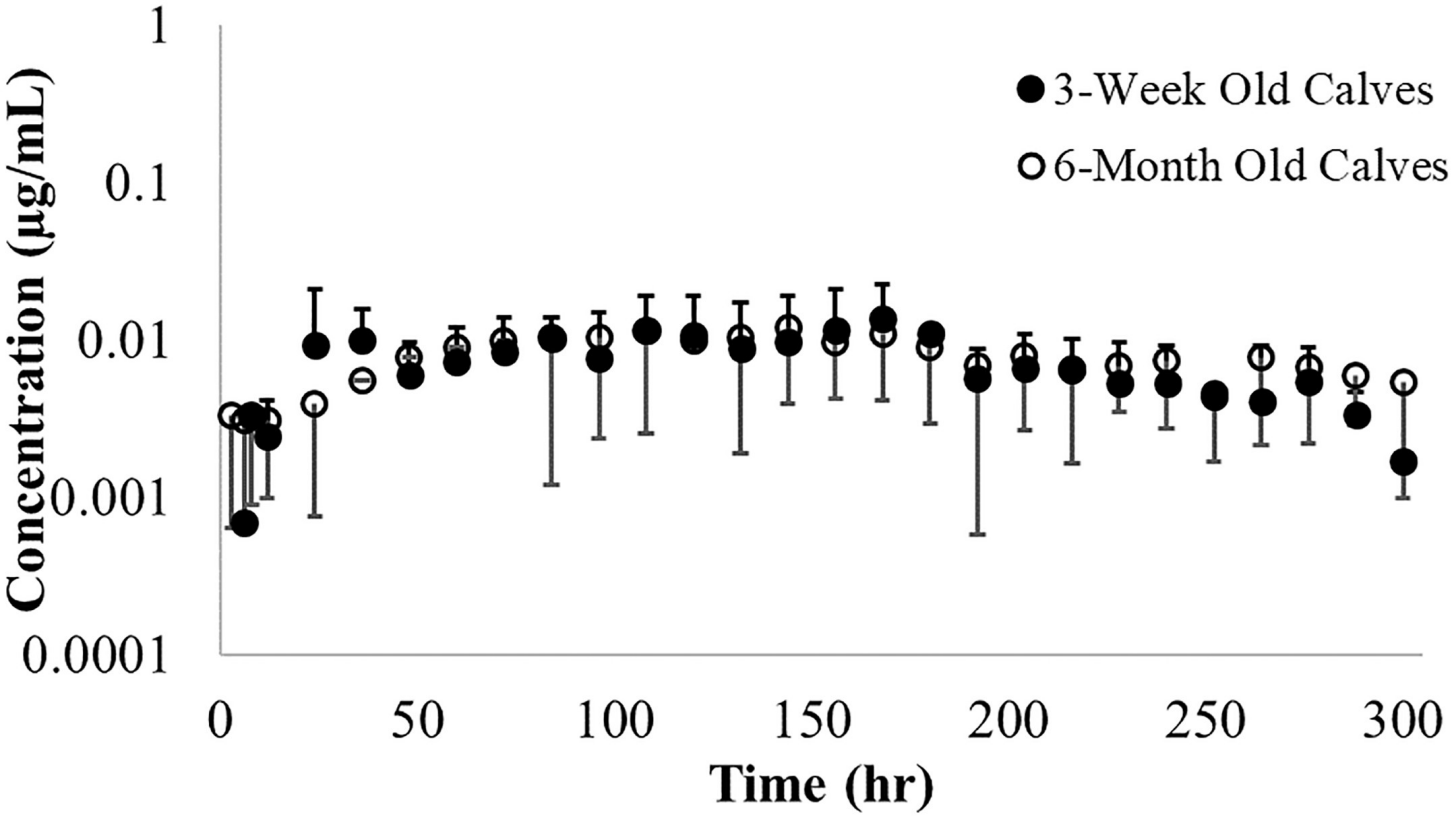
Mean interstitial fluid ± SD concentrations of tulathromycin. Tulathromycin was a after single S.C. injection of 2.5 mg/kg tulathromycin in 3-week old (solid circles) and 6-month old calves (open circles).

The T_max_ for ISF fluid occurred later than in plasma irrespective of calf age. Despite 6-month old calves showing increased variability in ISF concentrations as compared to 3-week old calves, there was no difference in the mean (±SD) concentration of tulathromycin in ISF samples from both groups of diseased calves concentrations at any time point (Fig 7).

#### Pulmonary epithelial lining fluid

**Fig 8:**
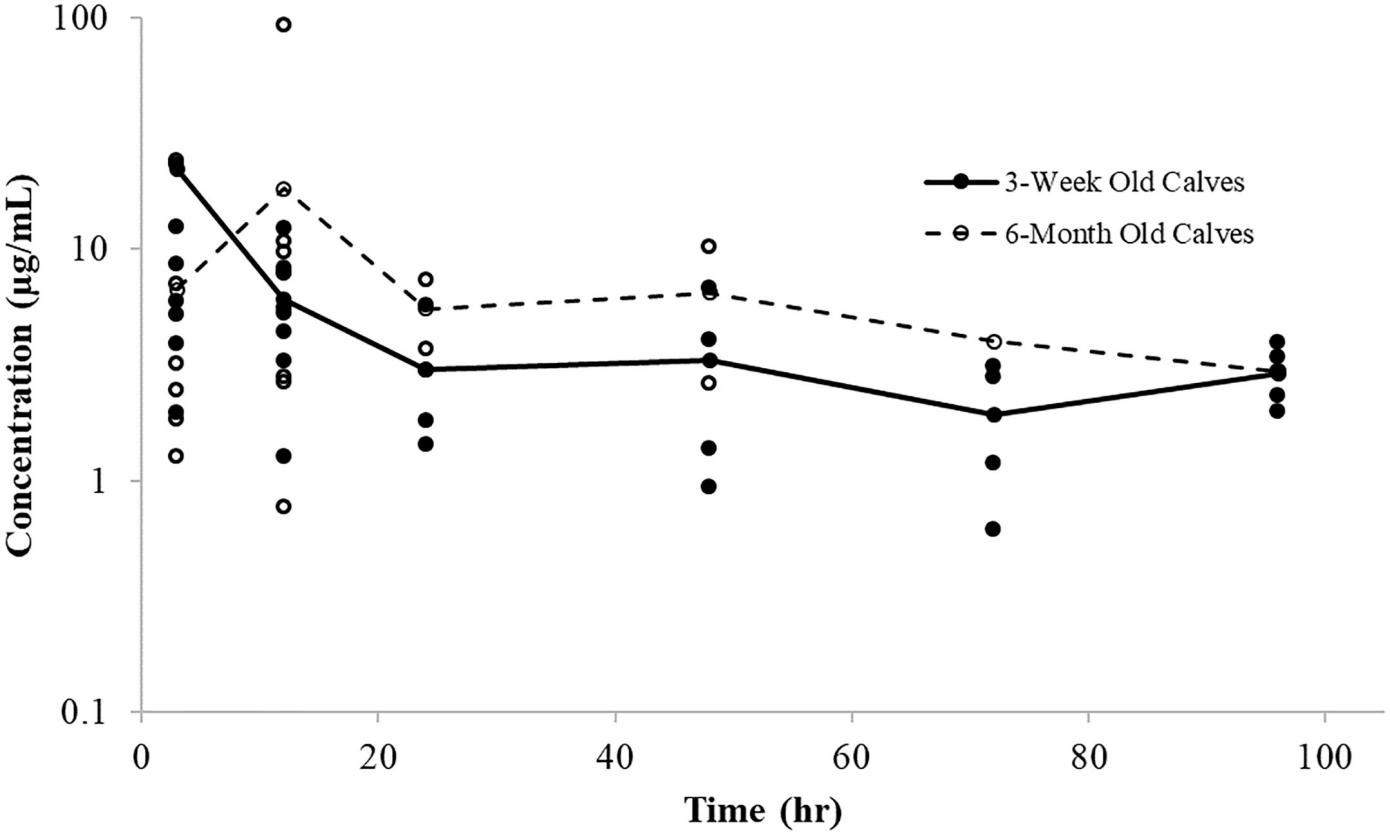
Individual PELF concentrations of tulathromycin. Lines represent average concentration over time for 3-week old calves (solid line) and 6-month old calves (dashed line).

The maximum concentration in PELF was noted to occur at 3 h post administration for 3-week old calves and around 12 hours in 6-month old calves, with estimated PELF concentrations far exceeding the concentrations seen in plasma and ISF at all time points. High variability in drug concentrations in PELF was seen in both age groups of calves administered tulathromycin (Fig 8).

## Discussion

One of the challenges to address was identifying an appropriate approach for analyzing the study data and the impact of the covariates (age and disease) on the PK of danofloxacin and tulathromycin in calves was selecting an appropriate approach for the data analysis. On the one hand, compartmental models are a simplification of the complex processes influencing the dose-exposure profiles and therefore are subject to bias due to model-misspecification. On the other hand, much information is lost through the use of simple non-compartmental (NC) methods. Moreover, for compounds such as tulathromycin (long residence time in the body although relatively rapid disappearance from the plasma) NC methods are inadequate for the questions that we are asking in this investigation. Therefore, the results of both methods of analysis are presented. Covariate analysis was subsequently generated using a NLME approach based upon the initial parameter estimates obtained using a two-compartmental model. Data collected in this study was analyzed by both methods.

### Respiratory disease model

Pneumonia is an important health problem in calves. In the current investigation, the calves showed clinical and physiological signs of respiratory disease after nebulization with the targeted bacterial pathogens. Changes in clinical parameters included an increase in animal heart rate, nasal and ocular discharge, and an increase in body temperature. During our clinical evaluation of these animals, we concluded that the thoracic ultrasound scores associated with disease resulting from use of the induction model shared some (although not identical to) the clinical and pathological manifestation of natural disease processes. The anterior–ventral lobar distribution of natural and experimental BRD is well recognized in cattle and that same pattern was noted on thoracic ultrasounds in the current experiment. Similar to previous disease models, the distribution of recognizable lesions in lung tissue is not uniform. The distribution of pathological processes may be influenced by the architecture of the lung parenchyma. Gravitational forces may account for some of the variability in the lesions noted after disease induction.

The concentration of *P*. *multocida* ranged from 1x10^9^ to 4x10^9^ but was not found to correlate with more severe clinical signs. A similar result was reported in experiments using lambs where differences of 3 logs in *M*. *haemolytica* A2 aerosols did not result correlate with the severity of the pneumonia [22]. This failure to determine any obvious correlation suggests that additional physiological factors can significantly contribute to the expression and severity of the BRD.

More severe clinical signs of respiratory disease were observed in 3-week old calves. Several pathophysiological changes could explain the more severe clinical signs in young calves as compared to 6-month old calves, including higher local bacteria dose in airways, lack of mature immunologic response and differences in physiological barriers in the alveoli-capillary membrane. All calves in this study received the same volume of nebulized bacteria. Young calves have smaller diameter airways, potentially leading to higher concentration of bacteria locally. The immune response in lungs of young calves in general is slower, less efficient, and much less focused than in older patients [23], which could lead to an infection that is less likely to be localized due to poor inhibition by the host defenses. Tissue damage from bacteria could predispose young calves to damage of the alveolar-capillary membrane and therefore an increase in membrane increased permeability. Bacteria and toxins in theory can cross this barrier, leading to endotoxemia, which has been shown to have critical effects on drug distribution and PK [24]. After birth, the formation and maturation of functional alveoli of the lungs that form the alveolar-capillary membrane continue, and immaturity of the barrier could explain more severe clinical disease in 3-week old calves [25]. Patients with acute pneumonia have demonstrated depressed myocardial function and dehydration from extracellular fluid shifts, leading to a decrease blood volume and effectively a decrease in cardiac output.

### Danofloxacin

We investigated the impact of experimental BRD on the PK and distribution of subcutaneously administered danofloxacin in two different ages of calves. Several authors have described plasma concentration-time relationships as well as determined concentrations in lung tissue, bronchial secretion and bronchial epithelium for danofloxcin in mature calves (with and without the presence of BRD), [6, 7, 26]. However, in our study, we further considered these relationships as a function of calf age. The results of the present investigation, as well as those of previous studies performed in healthy calves [19], were consistent with a two-compartment open model (Fig 1). In earlier plasma PK studies in healthy 6-month old beef calves, observed plasma C_max_ values ranged between 1.2 and 2.2 μg/mL [8]. In the current study involving diseased calves, the range of plasma C_max_ were higher (1.1–10.4 μg/mL for the 6-month old calves and 1.6–4.2 μg/mL for the 3-week old calves). Thus, there is a trend suggesting that higher C_max_ values in diseased animals. Importantly, age appeared to be more important than disease in terms of the peak concentrations.

Maximum danofloxacin concentrations in the bronchial secretions of ruminating calves infected with BRD was reported to occur by other investigators at approximately 2 hours post administration. The corresponding peak concentrations were about 6.9 μg/mL, with T_max_ values of about 2 hrs in the bronchial secretions [27]. Similarly in our study, the T_max_ in diseased 3-week and 6-month old calves in PELF averaged 2.4 and 2.0 hours. The C_max_ in PELF reached higher concentrations in 3-week old calves (4.54 μg/ml) as compared to 6-month old calves (1.61 μg/ml). Common respiratory pathogens, including *M*. *haemolytica* and *P*. *multocida*, are extracellular organisms, so determining concentrations at the site of infection in PELF may be more clinically relevant than lung tissue concentrations.

With regard to the plasma profiles, it has been previously noted that the presence of BRD could influence the T_1/2_ of danofloxacin [26]. Crossbred steer calves with acute pneumonia had a plasma T_1/2_ of approximately 6.3 h [26]. In the current study, we observed plasma T_1/2_ values of 30-hr for 3-week old calves and 29-hr for 6-month old calves. As observed in our previous studies in healthy calves, the longer time for depletion in the plasma concentrations reflected our ability to capture a second depletion phase. As compared to that reported in other investigations, the longer T_1/2_ estimated from the data generated in the present study may be attributed to the interdependence of the drug pKa, lipophilicity, metabolism pathways and elimination rate. It would be expected that as clearance decreases, (owing to disease processes), T_1/2_ would increase. However, this relationship only holds true when the disease process does not simultaneously decrease the Vd/F. For lipophilic drugs like danofloxacin, age (as well as pathophysiological shifts in body water and fat distribution) can be expected to influence the calculated T_1/2_, and any decrease in drug available in its unionized state due to pneumonia-induced respiratory acidosis (a change in the pH of the plasma and in other body tissues) could lead to a decrease in drug lipophilicity. This decrease in lipophilicity would decrease the movement of drug into the tissue compartment, thereby reducing the volume of distribution, and counteracting the effects of the decrease in drug clearance. In that regard, there is evidence that inflammation and disease can affect the acid-base exchange within the lung fluid [28]. Using a bronchoscopically-directed pH electrode, when compared to the pH observed in non-infected bronchi, the presence of pneumonia can significantly lower the pH of the infected bronchi [29]. Acidification of the PELF can also negatively impact the innate immune responses of the respiratory tract and impair the host response to pathogens and inflammation [30]. Since both danofloxacin and tulathromycin have multiple pKa values, the acidification of the PELF could potentially lead to an increase in tissue trapping, and an increase in local lung concentrations. However, this trapping would still need to overcome the impact of acidosis-associated changes in plasma pH. Thus, it is not surprising to find higher inter-individual variability in the PK of diseased as compared to normal healthy animals.

Total clearance of drugs is governed by blood flow to all eliminating organs, the unbound fraction and intrinsic clearance mechanisms. Unlike intrinsic clearance, which represents the ability of an organ to clear unbound drug and is typically unaffected by plasma protein binding [31–33], total drug clearance and total plasma drug concentrations can be influenced by the extent of plasma protein binding. Plasma protein binding in previous studies demonstrated minor differences between that observed in healthy 3-week versus 6-month old calves [19]. In disease, widely variable levels of a drug binding protein, alpha_1_-acid glycoprotein (AGP), can modify the total drug distribution of basic drugs [34]. For this discussion, the authors are specifically referring to changes in total drug concentrations and not to changes in the distribution of free drug concentrations.

It should be noted that other than albumin, there is very limited data quantifying tissue levels of AGP in cattle and therefore we will not consider the potential impact of changes in AGP on the free fraction in tissues and plasma.

The effect of age on danofloxacin CL/F values was significant. Assuming a similar fraction of drug absorbed from the injection site, CL/F values in 3-week old calves with respiratory disease (262.9 mL/kg/hr) was lower than in healthy 11–13 week old calves (578 mL/kg/h) [35] or in healthy 3-week old calves [19]. Maturation differences of CL/F values, as well as changes in blood flow and pathophysiology of clinical diseases in calves less than three weeks of age may impact the intrinsic clearance and elimination of danofloxacin. As compared to healthy 3-week old calves, 3-week old calves with respiratory disease had higher AUC values (23.2 μg*hr/mL and 32.1 μg*hr/mL, respectively) [19]. Danofloxacin is eliminated both by hepatic and renal clearance. Severe infection is associated with a decrease in organ blood flow. Renal blood flow is affected by any changes in cardiac output [36]. Thus, the observed lower CL/F values could be attributed, at least in part, to a reduction in the renal elimination of unchanged drug in diseased 3-week old calves.

In our previous studies performed in healthy calves, the ISF tended to be somewhat greater in the 3-week versus 6-month old calves, but the ratio of ISF/unbound plasma concentrations were markedly higher in the 6-month old versus 3-week old animals. In the current investigation, penetration of danofloxacin into the ISF did not differ significantly between the two age groups at any time point. However, a trend was noticed in the ISF:Plasma_Total_ ratio where there were differences noted in the shapes of the ratios in 3-week old calves vs 6-month old calves as a function of time (Fig 7). For the 3-week old calves, the ratios peaked at 24 h but their values remained lower than those seen in the 6-month old calves. In contrast, the ratios continued to decline over the duration of sampling period in 6-month old calves. While the reason for this disparity cannot be determined, we cannot exclude the possibility of age-associated differences in disease effects on plasma protein binding. Such age-associated difference could reflect the greater severity of disease seen in the 3-week versus 6-month old calves.

While these results are provocative, it is important to recognize several limitations to interpreting the ISF concentrations in calves with respiratory disease as compared to work previously published ISF data in healthy calves by other authors. First, previous studies using ultrafiltration probes in calves changed the sampling tube every 24 hours instead of every 12 hours, as was done in this study. Trials performed by our lab indicated that maximum fluid collected/hour occurred when the collection tubes were changed every 12 h instead of every 24 h. Second, the method used for capturing the ISF reflects an averaging of concentrations over a collection period. Accordingly, when estimated at the time of fluid sampling, the measured concentration will exceed the actual concentration of drug in the ISF at any given moment in time. Thus, the use of ultrafiltration tends to overestimate ISF concentrations at a point in time.

In the current investigation, danofloxacin PELF concentrations decreased considerably by 24 hours after administration, but remained higher than those seen in the plasma and the ISF. Danofloxacin concentrations in PELF collected from healthy calves showed lower concentrations in samples taken at 24 hours as compared to calves with induced respiratory disease at the same time points. Concentrations in PELF from calves with respiratory disease was significantly higher at 2 and 12 hours in 3-week versus 6 month-old calves. However, disease did not appear to influence the 2 hr PELF danofloxacin concentrations in the 3-week old calves [5.4 ± 6.5 μg/mL and 5.6 ± 3.4 μg/mL in healthy and respiratory disease calves respectively [19]]. However, at 24 hours, healthy 3-week old calves had lower danofloxacin concentrations (0.62 ± 0.86 μg/mL) when compared to calves with respiratory disease (2.3 ± 5.4 μg/mL). This could possibly have been of function of danofloxacin accumulation within the bronchial mucosa and lung tissues [7]. Similar disease effects were not observed at hr 24 in the 6-month old calves [3.6 ± 1.7 μg/mL) 1.6 ± 0.89 μg/mL in healthy and diseased calves, respectively [19].

An additional finding was that although concentrations of danofloxacin in the PELF were highly variable across both groups of calves, on the average, they were higher in the 3-week old calves as compared to that of the 6-month old calves at all-time points sampled. Although mean values differed, the magnitude of variability, particularly in diseased calves, rendered it difficult to draw conclusions. Rather, it would appear that the primary challenge was that of experimental whereby there was greater difficulty in obtaining reliable sampling of PELF in neonates as compared to mature calves. Furthermore, the sampling method used in this study may be overestimating fluoroquinolone concentrations in PELF [37]. Pathophysiological changes to the lung parenchyma and blood flow to affected lung lobes may also contribute to the observed high variability. Data from calves with no areas of pulmonary consolidation indicated that blood flow was significantly lower in the caudodorsal position of the left lung and in the caudodorsal and cranioventral positions of the right lungs as compared to other pulmonary locations [26].

The measurement of the danofloxacin in ISF and PELF is ideal as most respiratory pathogens seen in calves are extracellular bacteria. Collectively, these findings, along with previous studies in healthy calves, suggest that age and disease both impact danofloxacin concentrations in plasma, ISF and PELF. By measuring the drug concentration in the active sites of infection, a more accurate conclusion of clinical efficacy can be determined for specific populations of calves. However, even if that is not feasible, our results show that comparative blood levels were able to identify differences in drug PK as a function of age and disease.

### Tulathromycin

Plasma concentrations of macrolides such as tulathromycin in cattle are in general considerably low relative to the MIC of the extracellular respiratory pathogens for which they are used. A better measure of clinically relevant information would include measuring free/unbound drug concentrations at the site of infection. Studies of respiratory tract infections have demonstrated a role for white blood cells in the delivery of drug to the infection site. This is of importance for drugs, like tulathromycin, which concentrate in white blood cells and accumulate in lung tissue [38, 39].

We observed that irrespective of age, plasma tulathromycin concentrations were relatively low as compared to its concentrations in the PELF, was absorbed within less than 1 hr of dosing but declined slowly after SC administration (Fig 4). The median T_1/2_ was similar across both ages groups (116.5 (3-week old) and 91.6 hours (6-months old)). However, when compared to healthy calves, the mean T_1/2_ was nearly twice as long in the diseased calves (98.7 hrs vs 67.6 hr in diseased versus healthy 3-month old calves and 114.9 versus 44.4 hr in diseased versus healthy 6-month old calves [40]). Thus, disease markedly affected the duration of systemic tulathromycin exposure.

For macrolides, the use of blood levels for the assessment of dose when treating BRD does not directly mirror drug concentrations at the site of action. Unbound concentrations of tulathromycin in the ISF were therefore considered as a method for evaluating the movement of unbound drug from plasma throughout the body. Consistently, in healthy and diseased calves, concentrations in ISF were lower than were the plasma concentrations and were nearly superimposable across the two age groups. However, unlike that seen in healthy calves where the ISF/total plasma concentration ratios tended to be higher in the 6 month old calves during the initial sampling times, tulathromycin concentrations in the ISF were similar in 6-month old calves as compared to 3-week old calves with respiratory disease across the duration of the evaluation, suggesting that there was a greater ability of the drug to partition out of the blood of the diseased 3-week vs 6 month-old calves. Accordingly, the tulathromycin ISF: total plasma ratios tended to be higher in the 3-week versus 6-month old calves. While this interstudy difference could be due to the shorter sampling time duration in diseased calves (every 12 hours) vs healthy calves (every 24), other potential causes for this difference need to be considered. For example, in calves with respiratory disease, there is a potential for ionization in the ISF and PELF, due to respiratory acidosis and acidification of the PELF.

Several techniques have been described for estimating the PELF of macrolides including tissue homogenates, BAL, and bronchial microsampling [32, 41]. In contrast, the use of tissue homogenates does not allow for a differentiation between intracellular vs. extracellular drug concentrations or for the binding of drug to the tissues, therefore overestimating the active concentration of macrolides at the site of infection [42]. PELF is a reflection of the concentrations of the drug available at the site of bovine respiratory infections. Measurement of active/free drug in this compartment allows for the evaluation of drug exposure, and potential improvement of dosing strategies in different ages of cattle affected with BRD.

Tulathromycin concentrations in PELF were higher than concurrent plasma concentrations at all sampling times. PELF concentrations in diseased cattle did not differ between 3-week old and 6-month old calves and were typically similar in healthy and diseased calves. Given these limitations and uncertainties, PELF values should be considered as a rough approximation of pulmonary drug concentrations. However, this does not support concerns that concentrations estimated in healthy animals will be biased as compared to that in animals with BRD.

One 3-week old calf (Calf #34) administered tulathromycin after induction with aerosilizaed *P*. *multocida* demonstrated clinical signs of septicemia/endotoxic shock, including decreased heart rate, loss of suckle reflex, dehydration and cold extremities. Evaluating the calf’s plasma concentration profile revealed C_max_ concentrations 5x the average for other calves in the three-week-old group. The AUC was approximately double the reported mean. The CL/F for this calf was approximately 172 ml/hr/kg, about half of the reported mean for all 3-week old calves with respiratory disease. Other calves enrolled on this study showed signs of respiratory disease, but not septicemia/endotoxemia. Unlike typical BRD, septicemia indicates bacteria in the blood stream. Sepsis-induced liver hypoperfusion may result in a decreased clearance for high extraction ratio drugs [43]. These PK changes are consistent with previous studies evaluating the impact of fever and sepsis on PK parameters which demonstrated decreased clearance values and increased maximum plasma concentrations [24, 44].

## Conclusions

Data about the influence of disease on the pharmacology of drugs in pediatric calves are limited. The available studies mainly concentrate on plasma pharmacokinetics in healthy cattle, and largely ignore the effects of age and disease. Extrapolation of study results done in healthy adult cattle is difficult due to large variability in age-effects. In addition, studies in healthy animals fail to examine the underlying pathophysiological conditions and the pharmacokinetic characteristics of the drug concerned. Information on the effect of BRD is critical to estimate the extent of changes in pharmacokinetic parameters, facilitating efficacious drug use in calves.

The present study indicates that age, as well as disease, can affect the plasma pharmacokinetics of danofloxacin and tulathromycin in calves. However, it is important to recognize that therapeutic success is not only a function of drug exposure but also of the ability of the patient to launch an effective antibacterial response. The latter was not considered in this study, but we did observe that given the same mg/kg dose of danofloxacin and of tulathromycin, therapeutic success was maintained following artificial infection of both the 3-week and 6 month old calves.

While the overall influence of age appeared to be well characterized irrespective of whether one considered healthy or diseased animals for tulathromycin, this did not seem to be the case for danofloxacin. Macrolide antibiotics differ from danofloxacin in mechanism of action and PK parameters. Previously, age-associated differences in PK of tulathromycin of healthy calves included higher Vd/F in 6-month olds, as well as lower CL/F in 3-week old calves. Given the variability in data generated for tulathromycin, we conclude that these age-associated differences can be applied to both healthy and diseased calves. Clearly, this implies a need to consider both age and disease on a drug-by-drug basis and a need to better understand the effect of age and disease on the PK processes such as drug metabolism, transporter activity, and tissue perfusion.

## Supporting information

S1 Table Mean Respiratory scores and rectal temperatures for danofloxacin in 3-week old vs. 6-month old calves. Scores were taken prior to induction through hours post dosing. Ultrasound scores are median scores.
(DOCX)

S2 Table Mean Respiratory scores and rectal temperatures for tulathromycin in 3-week old vs. 6-month old calves. Scores were taken prior to induction through hours post dosing. Ultrasound scores are median scores.
(DOCX)

S1 Figures Goodness-of-fit plots for the final model of danofloxacin. (A) Observed concentrations (μg mL^–1^) versus individual predicted concentrations (μg mL^–1^) and (B) Observed concentrations (μg mL^–1^) versus population predicted concentrations (μg mL^–1^).
(JPG)

S2 Figures Goodness-of-fit plots for the final model of tulathromycin. (A) Observed concentrations (μg mL^–1^) versus individual predicted concentrations (μg mL^–1^) and (B) Observed concentrations (μg mL^–1^) versus population predicted concentrations (μg mL^–1^).
(JPG)
