# Supplementary material for: Impact of bovine respiratory disease on the pharmacokinetics of danofloxacin and tulathromycin in different ages of calves
Source: PLoS One. 2019 Jun 24;14(6):e0218864. doi: 10.1371/journal.pone.0218864 (PMC6590872; doi:10.1371/journal.pone.0218864)
Supplement: S1 Table — Scores were taken prior to induction through hours post dosing. Ultrasound scores are median scores. (DOCX) [file pone.0218864.s001.docx]

**S1 Table. Mean Respiratory scores and rectal temperatures or danofloxacin in 3-week old vs. 6-month old calves prior to induction through hours post dosing. Ultrasound scores are median scores.**

|  | | | |
| --- | --- | --- | --- |
|  | Rectal Temperature | Respiratory Score | Ultrasound Score |
| 3-Week Old Calves |  |  |  |
| Prior to Induction | 101.7 ⁰F | 1.6 | 0 |
| 0 hr | 102.0 ⁰F | 6.5 | 1 |
| 24 hr | 102.3 ⁰F | 3.7 | 2 |
| 72 hr | 101.9 ⁰F | 2.9 | 2 |
| 144 hr | 101.9 ⁰F | 2.5 | 2 |
| 6-Month Old Calves |  |  |  |
| Prior to Induction | 100.7 ⁰F | 1.1 | 0 |
| 0 hr | 102.6 ⁰F | 6.3 | 1 |
| 24 hr | 101.6 ⁰F | 2.8 | 1 |
| 72 hr | 101.4 ⁰F | 2.2 | 1 |
| 144 hr | 101.2 ⁰F | 0.8 | 1 |
