# Supplementary material for: Impact of bovine respiratory disease on the pharmacokinetics of danofloxacin and tulathromycin in different ages of calves
Source: PLoS One. 2019 Jun 24;14(6):e0218864. doi: 10.1371/journal.pone.0218864 (PMC6590872; doi:10.1371/journal.pone.0218864)
Supplement: S2 Table — Scores were taken prior to induction through hours post dosing. Ultrasound scores are median scores. (DOCX) [file pone.0218864.s002.docx]

**S2 Table. Mean Respiratory scores and rectal temperatures for tulathromycin in 3-week old vs. 6-month old calves prior to induction and hours after dosing. Ultrasound scores are median scores.**

|  | | | |
| --- | --- | --- | --- |
|  | Rectal Temperature | Respiratory Score | Ultrasound Score |
| 3-Week Old Calves |  |  |  |
| Prior to Induction | 102.7 ⁰F | 3.25 | 1.5 |
| 0 hr | 102.9 ⁰F | 7.4 | 2 |
| 24 hr | 102.1 ⁰F | 3 | 1.5 |
| 48 hr | 101.7 ⁰F | 2.9 | 1 |
| 72 hr | 101.7 ⁰F | 2.9 | 1 |
| 96 hr | 102.3 ⁰F | 2.5 | 1 |
| 144 hr | 102.2 ⁰F | 2.6 | 1.5 |
| 6-Month Old Calves |  |  |  |
| Prior to Induction | 102.1 ⁰F | 2.0 | 0.5 |
| 0 hr | 102.9 ⁰F | 6.6 | 1 |
| 24 hr | 102.3 ⁰F | 3.7 | 1 |
| 48 hr | 101.9 ⁰F | 3.6 | 1 |
| 72 hr | 101.9 ⁰F | 3.3 | 1 |
| 96 hr | 101.6 ⁰F | 2.4 | 2 |
| 144 hr | 101.1 ⁰F | 0.8 | 1.5 |
