# Supplementary figures and images for: Impact of bovine respiratory disease on the pharmacokinetics of danofloxacin and tulathromycin in different ages of calves
Source: PLoS One. 2019 Jun 24;14(6):e0218864. doi: 10.1371/journal.pone.0218864 (PMC6590872; doi:10.1371/journal.pone.0218864)

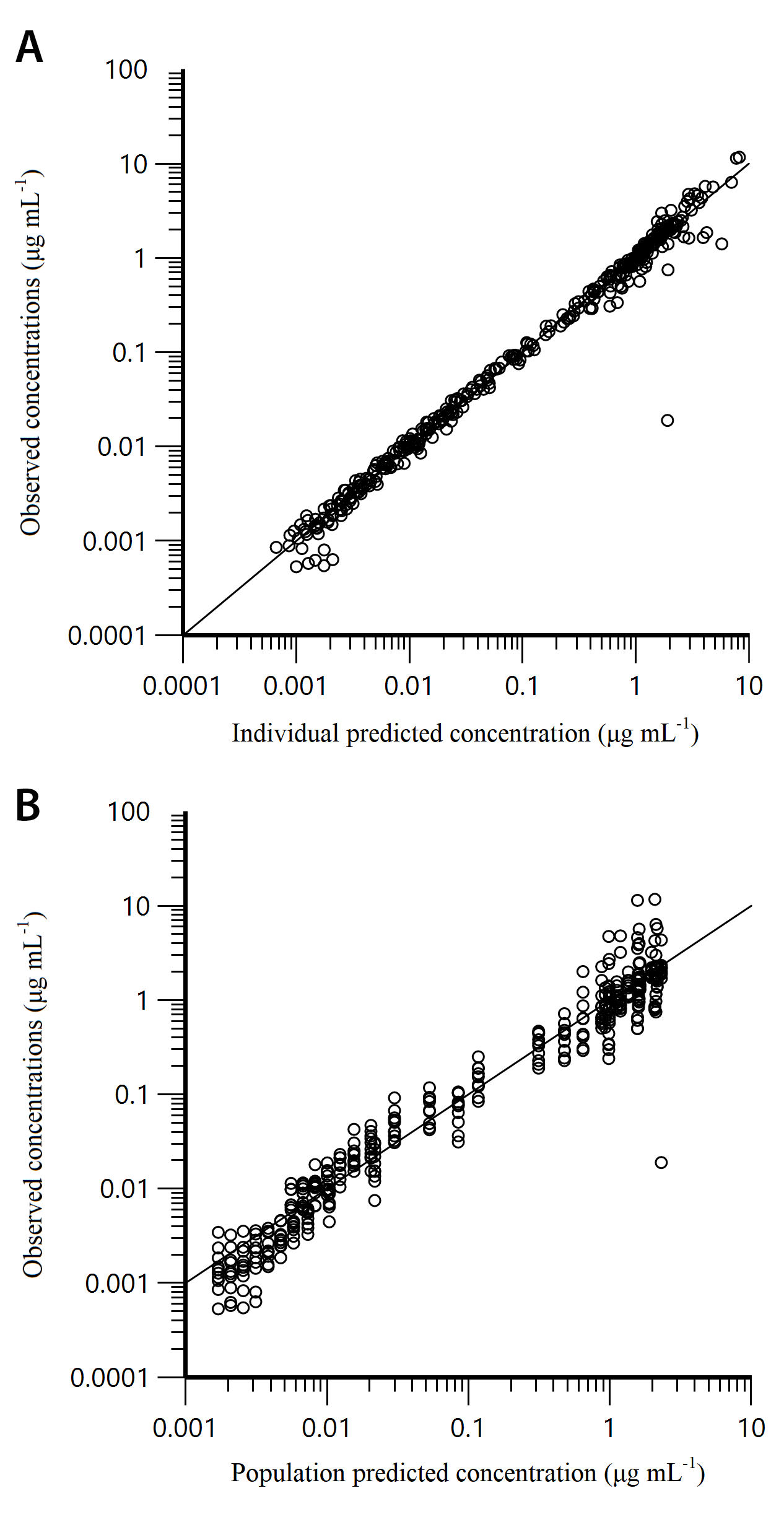

Supplement: S1 Figures — (A) Observed concentrations (μg mL–1) versus individual predicted concentrations (μg mL–1) and (B) Observed concentrations (μg mL–1) versus population predicted concentrations (μg mL–1). (JPG) [file pone.0218864.s003.jpg]

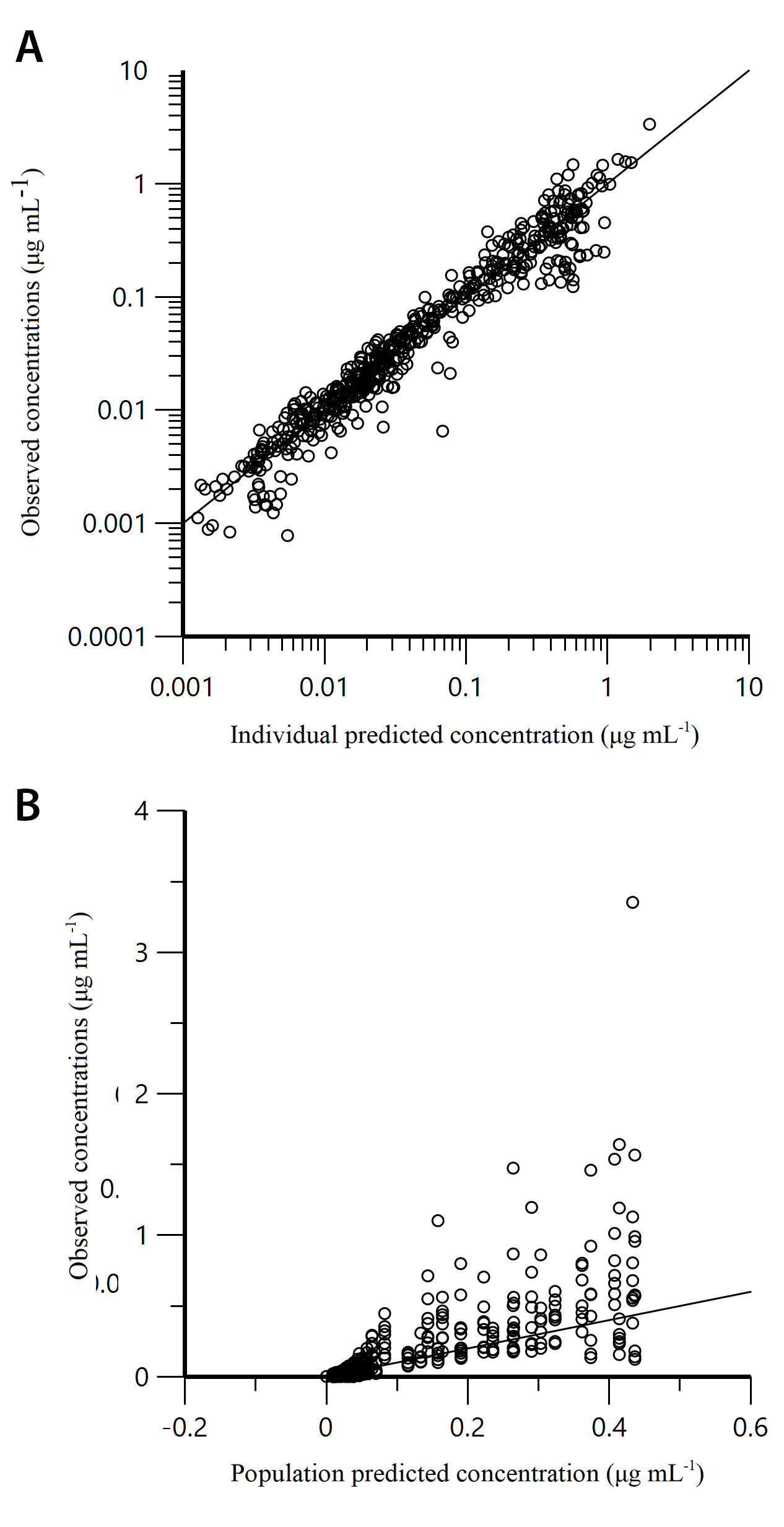

Supplement: S2 Figures — (A) Observed concentrations (μg mL–1) versus individual predicted concentrations (μg mL–1) and (B) Observed concentrations (μg mL–1) versus population predicted concentrations (μg mL–1). (JPG) [file pone.0218864.s004.jpg]
